# Models of service delivery for optimizing a patient’s first six months on antiretroviral therapy for HIV: an applied research agenda

**DOI:** 10.12688/gatesopenres.13159.1

**Published:** 2020-07-29

**Authors:** Sydney Rosen, Anna Grimsrud, Peter Ehrenkranz, Ingrid Katz

**Affiliations:** 1 School of Public Health, Boston University, 801 Massachusetts Ave, 3rd fl, Boston, MA, 02118, USA; 2 Health Economics and Epidemiology Research Office, Department of Internal Medicine, School of Clinical Medicine, Faculty of Health Sciences, University of the Witwatersrand, Johannesburg, South Africa; 3 HIV Programmes & Advocacy, International AIDS Society, Cape Town, South Africa; 4 Bill & Melinda Gates Foundation, Seattle, WA, USA; 5 Department of Medicine, Brigham and Women's Hospital, Boston, MA, USA; 6 Harvard Medical School, Boston, MA, USA; 7 Center for Global Health, Massachusetts General Hospital, Boston, MA, USA

**Keywords:** HIV, antiretroviral therapy, differentiated models, retention, Africa

## Abstract

Differentiated models of service delivery (DSD models) for HIV treatment in sub-Saharan Africa were conceived as a way to manage rapidly expanding populations of experienced patients who are clinically “stable” on antiretroviral therapy (ART). Entry requirements for most models include at least six months on treatment and a suppressed viral load. These models thus systematically exclude newly-initiated patients, who instead experience the conventional model of care, which requires frequent, multiple clinic visits that impose costs on both providers and patients. In this open letter, we argue that the conventional model of care for the first six months on ART is no longer adequate. The highest rates of treatment discontinuation are in the first six-month period after treatment initiation. Newly initiating patients are generally healthier than in the past, with higher CD4 counts, and antiretroviral medications are better tolerated, with fewer side effects and substitutions, making extra clinic visits unnecessary. Improvements in the treatment initiation process, such as same-day initiation, have not been followed by innovations in the early treatment period. Finally, the advent of COVID-19 has made it riskier to require multiple clinic visits. Research to develop differentiated models of care for the first six-month period is needed. Priorities include estimating the minimum number and type of provider interactions and ART education needed, optimizing the timing of a patient’s first viral load test, determining when lay providers can replace clinicians, ensuring that patients have sufficient but not burdensome access to support, and identifying ways to establish a habit of lifelong adherence.

## Disclaimer

The views expressed in this article are those of the author(s). Publication in Gates Open Research does not imply endorsement by the Gates Foundation.

## Introduction

As countries around the world strive to reach global targets for HIV, including starting and retaining 95% of those diagnosed with HIV on antiretroviral treatment (ART), one of the most promising recent strategies has been the advent of “differentiated service delivery” (DSD) models for providing treatment. DSD models are approaches to delivering ART that adjust the location, timing, provider, or service delivered with the goals of making care more patient-centered, supporting treatment outcomes, and making HIV programs more efficient ^1^. In sub-Saharan Africa, common DSD models include medication pickup points outside of health facilities, “fast-track” stations at clinics for patients to obtain medication refills without waiting in the regular clinic queue, and group models such as adherence clubs that allow patients to receive refills, adherence counseling, and other services together ^2^. Limited existing data suggest that most of these models either sustain or improve treatment outcomes ^3^ and succeed in making treatment more convenient and/or less expensive for patients, by bringing services closer to their homes and reducing waiting times ^4^.

Because DSD models were originally conceived as a way to manage rapidly expanding populations of experienced patients who are clinically “stable” on ART, most do not cater to newly-initiated patients. Entry requirements for most models include both a minimum number of months on treatment—usually six or 12—and a report of a suppressed viral load or comparable evidence of treatment success. In a recent review of published descriptions of DSD models in Africa, more than 70% required at least six months on ART for DSD entry, while 84% explicitly limited participation to “stable” patients ^5^.

As a result of the requirement for clinical stability, newly-initiated patients are systematically excluded from DSD models during their first six (or 12) months on ART, no matter their conditions, needs, or viral load. They instead experience the traditional or conventional model of care for newly initiated patients, which has changed relatively little in the past decades. Although there has been some streamlining, most national guidelines continue to call for monthly visits to a clinical facility for the first six months of treatment, with only short (one to two month) drug refills.

In this open letter, we argue that this conventional model of care for a patient’s first six months on ART may no longer be appropriate, for several reasons. First, the highest rates of treatment discontinuation are in the first six-month period after treatment initiation. This has been the case since the earliest published estimates of retention rates ^6, 7^ and remains the case now ^8^. Among patients who initiated ART in the last quarter of 2018 in South Africa, for example, 30% were reported as lost to follow up by six months ^9^. In Zimbabwe, retention in care improved significantly between 2010 and 2015 except for patients in their first six months ^10^. Beyond the impact on individual morbidity and mortality, early losses from care are associated with internalized stigma, leading to social isolation, fear of disclosure, and discrimination, which potentially compound the inherent challenges in returning to care ^11– 13^.

Second, while the model of care for newly-initiated patients has not changed over time, the patients and the drug regimens they are taking have. CD4 counts at treatment initiation have risen steadily since the advent of universal treatment eligibility ^14^ even as the proportion of patients with advanced disease has remained constant ^15^ and “re-initiation” of those who have previously interrupted care has become more common ^16^. Most newly initiating patients do not need additional clinical care after ART initiation. In a recent study in South Africa, for example, 86% of patients were considered clinically well enough for same-day ART initiation, without the need for additional care ^17^. Current antiretroviral regimens are also better tolerated than previous first-line regimens, requiring fewer drug changes ^18^.

**Figure 1.**
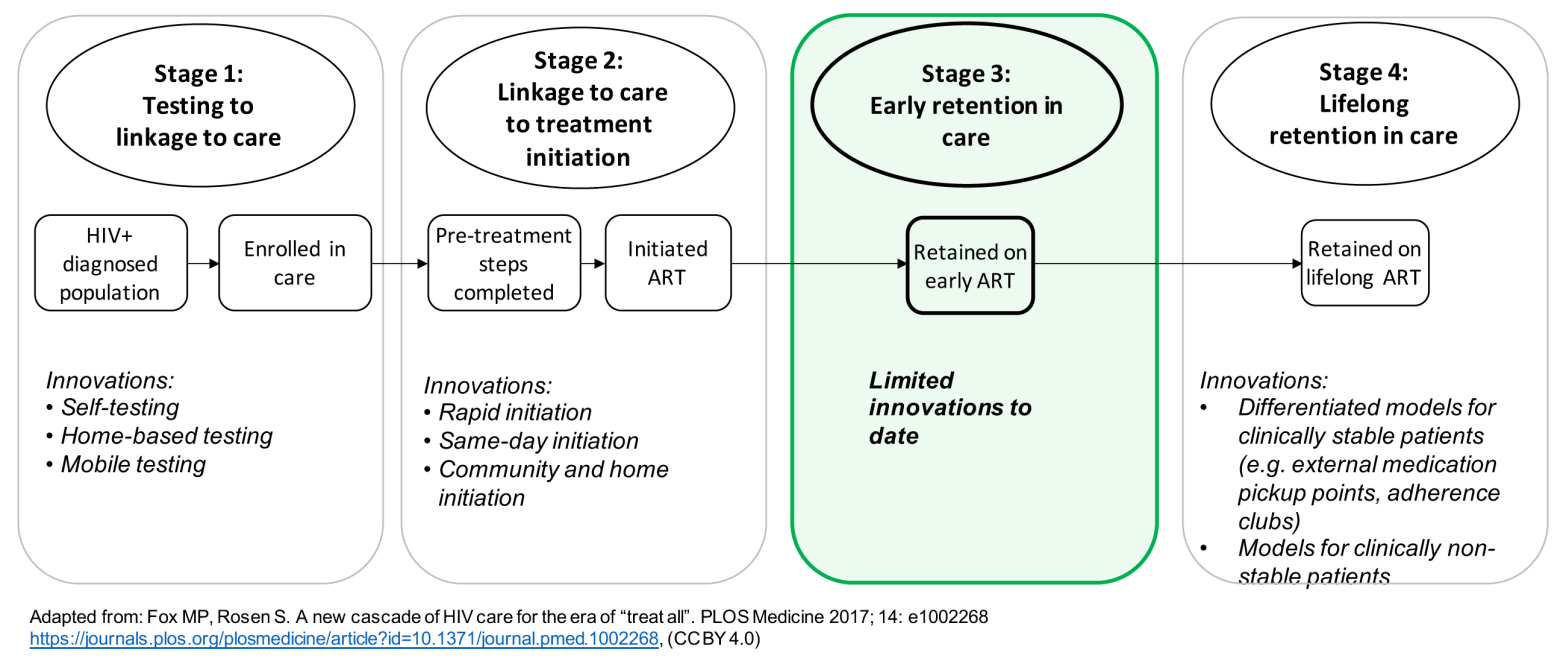
Innovations in service delivery along the HIV treatment cascade. Adapted from 23 under a CC BY 4.0 license.

Third, to date, efforts at “patient-centeredness” have largely bypassed the first six months on ART. As illustrated in Figure 1, innovations to make treatment more accessible in terms of time and transport costs and more satisfactory for patients do not extend to new initiators.

Fourth, while treatment guidelines have changed little for this period, procedures for initiating ART have evolved with the advent of rapid and same-day initiation ^19^. New initiators no longer undergo multiple counseling and education sessions before initiation. As a result, it is possible—though studies conflict on this issue ^8, 20^—that patients may be less prepared in advance for the reality of daily medication adherence and regular prescription refills and thus be more likely to disengage from care early on.

Fifth, the global epidemic of COVID-19 is highlighting the potential risks of exposure to SARS-CoV-2 at healthcare facilities both for patients and staff and, due to physical distancing ordinances and lockdowns, access to ART is more difficult and expensive for patients to achieve ^21, 22^.

In response to all these factors—burdensome, dated procedures for the first six months of therapy, rapid ART initiation, patients’ improving health condition at presentation, high attrition during this period, and COVID-19—reconsideration of how to deliver ART during the first six months is warranted and overdue. In early March 2020 we convened a half-day roundtable to explore what one or more optimized models of service delivery might look like for this period. Participants included clinicians, epidemiologists, economists, HIV program implementers, funders, and advocates. After reviewing the data cited above, the roundtable focused on the specific requirements of the first six months on ART, with the discussion divided into patients’ clinical and non-clinical needs. A preliminary research agenda was then proposed for developing new differentiated service delivery models for the first six months on ART. A version of that agenda further developed by the authors is reported here.

## Stratifying patient populations

A first consideration for improving care during the first six months was that patients in this population are not homogeneous. In addition to varying in age, sex, and other demographic and socioeconomic characteristics, patients differ at treatment initiation in terms of their clinical characteristics and their prior exposure to ART. While multiple criteria for segmenting populations were proposed, three major categories of patients were ultimately identified, based on patients’ status at initiation: 1) clinically well, ART-naïve initiators (new initiators); 2) clinically well, ART-experienced initiators (re-initiators); and 3) people presenting for initiation or re-initation with advanced disease. We note that “clinically well” is open to interpretation and likely includes patients who are mildly symptomatic but ambulatory and not critically ill, while the advanced disease category, following WHO guidance ^19^, may include patients who appear well but have low CD4 counts. We also are aware that a patient’s status as naïve or re-initiating is often based on self-report, as most countries do not have data systems that allow real-time monitoring of prior treatment.

Each of these three categories of patients is likely to have distinct clinical and non-clinical needs during the first six months. New initiators do not have experience with ART and should be supported with treatment education. Re-initiators, by definition, have already encountered at least one barrier to remaining in care; that barrier may well re-emerge if it is not addressed directly. Re-initiators can further be stratified by the timing of their prior disengagement in care and adherence patterns ^24^. People with advanced disease, in addition to potentially being in poor health requiring immediate medical care, are typically presenting late, suggesting that they too may face obstacles that led them not to seek care sooner ^25^. In this paper we focus primarily on the first category, new initiators, for whom the standard of care has changed little in recent years.

## Clinical and non-clinical requirements

Priority research questions are presented in Table 1. The first set of questions pertains to the clinical needs of patients during their first six months on treatment. During this period, most countries require at least two, and up to six, post-initiation clinic visits when a patient is required to be seen by a clinician, in addition to receiving drug refills and adherence counseling. Most new and re-initiators do not require any clinical interventions during this period, however, provided that any conditions present at ART initiation—opportunistic infections, side effects, or other acute concerns—were addressed by the clinician responsible for ART initiation, as part of the initiation process. Re-initiators who previously stopped ART due to side effects may also be prescribed more appropriate regimens.

**Table 1.** Priority research questions for developing models of care for the first six months on ART.

Question	Rationale and potential data sources or study designs
***Questions pertaining to clinical requirements***	
*1. Is a routine clinic visit required between ART* *initiation and month 6, and if so, exactly what* *procedures, tests, or other activities should it* *entail and when should it take place?*	The necessity of a routine clinical visit in the first six months on ART for most patients is unclear. Existing data that might answer this question are medical records for patient cohorts in this period that contain details of visits, clinicians seen (e.g. doctor, nurse, clinical officer), and changes made as a result of the visit. If most routine visits result in no changes to the patient’s regimen or other procedures or behaviors, then the visit should be dropped if it is not justified in some other way—for example, the patient who has a co-morbidity that requires more frequent consultation management or is in a risk group that requires special attention.
*2. What is the optimal timing for a patient’s first* *on-treatment viral load test? (Note that this does* *not refer to a baseline or initiation viral load.)*	Since viral suppression is often one of the key requirements for being considered clinically “stable” on ART—and is also itself the primary goal of ART—achieving and confirming suppression as soon after initiation as possible is considered desirable. Conducting a test at 6 months, which is the norm in most countries, is likely considerably later than is optimal, given the shorter time required for suppression with current recommended first-line drug regimens ^26, 27^. Roundtable participants were mixed as to whether three or four months after initiation would be optimal, but there was no support for waiting for six months and thereby potentially delaying both eligibility for a less-intensive model of care and the patient’s own peace of mind. Existing data to answer this question include both observational and trial data on time to viral suppression for different patient populations and drug regimens. Such data are currently being reviewed by a WHO guideline committee to update guidance on timing of the first viral load. Both CD4 count at initiation and ARV regimen matter to this issue. We note, for example, that in the ADVANCE trial of a first line regimen containing dolutegravir, 97% of patients were suppressed (viral load less than 1000 copies/milliliter) four weeks after initiation ^27^.
*3. What are the main reasons for unscheduled* *visits in this period, and how can patients* *best be prepared to recognize conditions that* *require clinical care?*	The main risk to eliminating or reducing routine clinic visits is that patients who need care will not obtain it, either because they do not recognize the symptoms, believe that they should or must wait for a routine appointment, or are unable to access the clinic for logistical or other reasons. The first two of these constraints can be addressed through better patient preparation and education at the time of ART initiation. The third is not unique to care on demand—if a patient cannot afford to access the clinic on demand, it is likely that routine appointments will also be missed. Other support mechanisms are needed in this case. Existing data that could be used to ensure that patients seek care when needed are data sets that report reasons for unscheduled clinic visits. While routine EMR data sets in many countries often report patient condition incompletely, data from trials, adherence studies, and carefully observed cohorts should suffice.
*4. Can a lay provider and/or virtual contacts* *adequately assess clinical condition during the* *early treatment period and replace in-person* *visits with a trained clinician?*	Rather than requiring a routine clinic visit after, for example, one month on ART, a trained community health worker or another lay provider may be able to use a checklist of symptoms to screen newly initiated patients by telephone or during a home visit, avoiding the need for an in-person visit for most of these patients. It is even possible that this could be done electronically in some settings, using an on-line software application (chatbot) to send and receive text or WhatsApp messages via patients’ phones. Similarly, if contact with a trained clinician is considered essential, video platforms (telemedicine) might make it possible for in-person visits to be replaced with digital consultations in some settings. Research is needed to determine if existing cadres of lay health workers can implement a process like this safely and can correctly distinguish between patients who should be referred for additional care and those who should not and if and where available bandwidth, device access, and software could support more use of electronic, rather than in-person, care ^28^.
***Questions pertaining to non-clinical requirements***	
*5. How much non-clinical interaction with* *healthcare providers is optimal between ART* *initiation and eligibility for an existing, stable-* *patient model of care?*	Individual patients vary widely in their needs and expectations for contact with lay or professional healthcare providers. As mentioned above, roundtable participants thought that at least one interaction during the first month after initiation is essential, both to confirm that the patient is responding well to treatment and to establish a pathway for communication with which the patient is comfortable. Beyond this interaction in month 1, research is needed to know which patients would benefit from additional contact; when, where, how, and with whom such contact should be; and the best pathways to ensure that patients who would benefit do receive such support.
*6. What is the best way to ensure adequate HIV* *and ART knowledge (education) among newly* *initiated patients?*	Roundtable participants noted that “treatment education” has diminished in recent years, as the process of ART initiation has accelerated, and believed that a lack of understanding of HIV and ART is partly responsible for the high rates of early attrition observed. Data are sparse, though, on what encompasses sufficient understanding or how best to ensure it, without burdening patients with additional clinic visits or delaying treatment initiation. Research on different approaches to delivering treatment education in a manner in which it is likely to be retained is also needed.
*7. Are there specific additional services or* *approaches that would help patients establish a* *long-term habit of ART adherence?*	The early treatment period is critical not only to rebuild immune function and achieve viral suppression but also to instill in new patients a lifelong habit of adherence to treatment. Roundtable participants recognized that there is a behavioral science literature on habit formation that may offer insights into how to strengthen antiretroviral adherence, and that this literature should be accessed to develop potential early interventions to improve long-term outcomes. Research patient preferences for services, for example using discrete choice experiments, is also needed.
*8. What is the role of disclosure in promoting* *early retention, and should there be explicit* *support for disclosure during this period?*	Existing research indicates that fear of disclosure and discrimination are among the causes of early losses from treatment ^11– 13^. It is unclear, though, whether support to patients for disclosure and/or other interventions to reduce barriers such as stigma and interpersonal violence are essential during the first six months on treatment and, if so, for whom, by whom, and how they should be delivered. For patients who do need such support, intervention during the early treatment period may be a prerequisite to achieving retention on ART. Various peer-based interventions have been tried ^29, 30^, but research is still needed on an optimal approach to delivering disclosure support and, importantly, to determine which patients would benefit from such support, without burdening those who would not.
*9. Should patients opt in, opt out, or “qualify* *out” of components of specific non-clinical* *aspects of support, and how should this be* *managed?*	Ideally, treatment programs will be able to offer patients a choice of ART retention-related services tailored to the early treatment period, such as adherence counseling, home visits, and virtual interactions. It is unclear if such services should follow an “opt-in”,“opt-out” or “qualify out” model to achieve retention targets and increase patient satisfaction. By “qualify out”, we mean that both the provider and the patient should be confident that the patient has sufficient treatment knowledge, understands what HIV- and ART-related conditions should trigger a phone call or clinic visit, and has access to a working phone. This question is related to the broader issue of how responsibility for retention on ART should be allocated between providers and patients and the extent to which patients should be offered choices in service delivery.
*10. What additional or different non-clinical* *services are needed for re-initiators?*	By definition, ART re-initiators have already experienced one or more barriers to retention in care that were sufficiently serious to cause disengagement. Simply re-initiating ART without identifying and addressing those barriers seems unlikely to achieve long-term retention for most of these patients. Research is needed on how providers can most effectively support this process without creating even more barriers, for example by requiring additional clinic visits for adherence counseling.

The other major set of questions, also shown in Table 1, pertained to the emotional, social, and other non-clinical needs of patients in their first six months. While participants were generally comfortable with the notion that frequent (monthly) clinic visits and clinical consultations are not essential for most patients after initiation, there was a consensus that some interaction with a care provider during at least the first month on ART, even if merely a text message exchange with a community health worker, remains important to securely engage patients in care for the short and long term. Beyond that, ensuring that patients have adequate information about HIV and ART and access to on-demand emotional and social support, virtually or in person, was thought sufficient.

## Other considerations

In addition to the research questions specified in Table 1, several issues were raised that were considered important for efforts to develop new models of care for the first six months. First, the quality of the ART initiation process is crucial to determining early outcomes on treatment. While same-day and rapid initiation are effective in reducing pre-initiation loss to follow up, poor quality in the initiation process will have the opposite effect on retention after initiation. At the point of treatment initiation, providers must address both acute and chronic co-morbidities, help patients identify potential adherence barriers in advance, and convey sufficient information about ART and available support services that patients can effectively manage their own care going forward. More attention should thus be paid to the quality of the initiation process, as well as its speed.

Second, any new models of care proposed for the early treatment period must be able to withstand incomplete or poor fidelity to guidelines and unreliable access to resources. Complicated models that will be effective only if providers closely follow guidelines and/or have access to items that may suffer stock-outs are not likely to succeed. The same conditions should apply to research methods used in evaluations of new models of care; study designs must be robust to non-compliance with guidelines and secular changes that affect both intervention and comparison groups.

Third, the possibility was raised of triaging patients to more or less intensive retention support at the time of ART initiation, based on patient characteristics. If higher and lower risk patients could be identified at the start, providers could potentially offer tailored support plans to those at higher risk, while allowing those at lower risk to proceed with less intervention. Unfortunately, at this point, data on practical predictors of poor retention do not exist, despite efforts to create risk indices ^31^. Further research in this area may also be of value.

Finally, patients who present with advanced disease require different approaches than the majority who have no or mild symptoms. Clearly those who are acutely ill need immediate care, regardless of the burden it imposes. Current guidelines and practices, however, generally require even asymptomatic patients with low CD4 counts to make additional clinic visits. If these patients have advanced disease because they face challenges in seeking treatment, and thus presented late, simplifying care during the first six months may be even more important for them than for healthier patients.

## Conclusion: the role of COVID-19

The advent of the COVID-19 pandemic is rapidly changing national guidelines and practice in HIV treatment. Many countries have begun to extend the duration of ART refills at treatment initiation and reduce clinical encounters during the early treatment period. These changes are occurring rapidly, and implementation is likely uneven across regions and individual facilities and programs. Evaluation of the outcomes of these measures is essential, as they provide a natural experiment with different approaches to initiation and early treatment. As the COVID-19 crisis recedes, data on steps that were taken and their effects on patient welfare will be a critical source of information for improving the early treatment algorithm in the future. These data will add to our evidence base on what services can effectively be provided outside of clinic facilities or remotely, and under what circumstances.

## Data availability

No data are associated with this article.

## References

[1] Grimsrud A Bygrave H Doherty M: Reimagining HIV service delivery: the role of differentiated care from prevention to suppression. *J Int AIDS Soc.* 2016;19(1):21484. 10.7448/IAS.19.1.21484 27914186 PMC5136137

[2] Grimsrud A Barnabas RV Ehrenkranz P: Evidence for scale up: The differentiated care research agenda. *J Int AIDS Soc.* 2017;20(Suppl 4):22024. 10.7448/IAS.20.5.22024 28770588 PMC5577722

[3] Long L Kuchukhidze S Pascoe S: Retention in care and viral suppression in differentiated service delivery models for HIV treatment in sub-Saharan Africa: a rapid systematic review. *Preprints.* 2020;1–21. 10.20944/preprints202005.0314.v1 PMC7696000 33247517

[4] Kuchukhidze S Long L Pascoe S: Patient benefits and costs associated with differentiated models of service delivery for HIV treatment in Sub-Saharan Africa. AMBIT Report No. 01. Unpublished. Boston: Boston School of Public Health,2019 Reference Source

[5] AMBIT Project: Eligibility criteria for differentiated service delivery models of hiv treatment in Sub-Saharan Africa. Unpublished. Boston: Boston School of Public Health,2020 Reference Source

[6] Rosen S Foxn MP Gill CJ: Patient retention in antiretroviral therapy programs in sub-Saharan Africa: a systematic review. *PLoS Med.* 2007;4(10):e298. 10.1371/journal.pmed.0040298 17941716 PMC2020494

[7] Fox MP Rosen S: Retention of adult patients on antiretroviral therapy in low- and middle-income countries: systematic review and meta-analysis 2008–2013. *J Acquir Immune Defic Syndr.* 2015;69(1):98–108. 10.1097/QAI.0000000000000553 25942461 PMC4422218

[8] Lilian RR Rees K McIntyre JA: Same-day antiretroviral therapy initiation for HIV-infected adults in South Africa: Analysis of routine data. *PLoS One.* 2020;15(1):e0227572. 10.1371/journal.pone.0227572 31935240 PMC6959580

[9] Diseko L Overmeyer R: South Africa DSD update. Johannesburg: CQUIN Annual Meeting, November 12,2019 Reference Source

[10] Makurumidze R Mutasa-Apollo T Decroo T: Retention and predictors of attrition among patients who started antiretroviral therapy in Zimbabwe’s national antiretroviral therapy programme between 2012 and 2015. *PLoS One.* 2020;15(1):28–42. 10.1371/journal.pone.0222309 31910445 PMC6946589

[11] Katz IT Ryu AE Onuegbu AG: Impact of HIV-related stigma on treatment adherence: systematic review and meta-synthesis. *J Int AIDS Soc.* 2013;16(3 Suppl 2):18640. 10.7448/IAS.16.3.18640 24242258 PMC3833107

[12] Rueda S Mitra S Chen S: Examining the associations between HIV-related stigma and health outcomes in people living with HIV/AIDS: a series of meta-analyses. *BMJ Open.* 2016;6(7):e011453. 10.1136/bmjopen-2016-011453 27412106 PMC4947735

[13] Pantelic M Casale M Cluver L: Multiple forms of discrimination and internalized stigma compromise retention in HIV care among adolescents: findings from a South African cohort. *J Int AIDS Soc.* 2020;23(5):e25488. 10.1002/jia2.25488 32438498 PMC7242009

[14] IeDEA and COHERE Cohort Collaborations, Anderegg N Panayidou K: Global trends in CD4 cell count at the start of antiretroviral therapy: collaborative study of treatment programs. *Clin Infect Dis.* 2018;66(6):893–903. 10.1093/cid/cix915 29373672 PMC5848308

[15] Ford N Meintjes G Calmy A: Managing advanced HIV disease in a public health approach. *Clin Infect Dis.* 2018;66(Suppl_2):S106–S110. 10.1093/cid/cix1139 29514232 PMC5850613

[16] Yonga P Kalya S Lynen L: Temporary disengagement and re-engagement in human immunodeficiency virus care in a rural county serving pastoralist communities in Kenya: A retrospective cohort study. *Int Health.* 2019;12(2):95–100. 10.1093/inthealth/ihz049 31227824 PMC7057135

[17] Maskew M Brennan AT Fox MP: A structured algorithm for same-day art initiation: SLATE II trial primary outcomes. Abstract 1070, CROI 2020, March 8-11,2020 Reference Source

[18] Brennan AT Bor J Davies MA: Medication side effects and retention in HIV treatment: A regression discontinuity study of tenofovir implementation in South Africa and Zambia. *Am J Epidemiol.* 2018;187(9):1990–2001. 10.1093/aje/kwy093 29767681 PMC6118076

[19] World Health Organization: Guidelines for managing advanced HIV disease and rapid initiation of antiretroviral therapy. Geneva: World Health Organization,2017. 29341560

[20] Siedner MJ Lankowski A Haberer JE: Rethinking the ‘“Pre”’ in Pre-Therapy Counseling: No Benefit of Additional Visits Prior to Therapy on Adherence or Viremia in Ugandans Initiating ARVs. *PLoS One.* 2012;7(6):e39894. 10.1371/journal.pone.0039894 22761924 PMC3383698

[21] Ndaliso C: Thousands of HIV-positive patients miss hospital appointments amid coronavirus lockdown. *IOL.* 30 Apr 2020 Reference Source

[22] Molelekwa T: Barriers to ARV access during lockdown. *Health-e News.* 2020 Reference Source

[23] Fox MP Rosen S: A new cascade of HIV care for the era of “treat all”. *PLoS Medicine.* 2017;14:e1002268 10.1371/journal.pmed.1002268 28399160 PMC5388465

[24] Mody A Eshun-Wilson I Sikombe K: Longitudinal engagement trajectories and risk of death among new ART starters in Zambia: A group-based multi-trajectory analysis. *PLoS Med.* 2019;16(10):e1002959. 10.1371/journal.pmed.1002959 31661487 PMC6818762

[25] Ahmed S Autrey J Katz IT: Why do people living with HIV not initiate treatment? A systematic review of qualitative evidence from low- and middle-income countries. *Soc Sci Med.* 2018;213:72–84. 10.1016/j.socscimed.2018.05.048 30059900 PMC6813776

[26] Kerschberger B Boulle AM Kranzer K: Superior virologic and treatment outcomes when viral load is measured at 3 months compared to 6 months on antiretroviral therapy. *J Int AIDS Soc.* 2015;18(1):20092. 10.7448/IAS.18.1.20092 26403636 PMC4582072

[27] Venter WDF Moorhouse M Sokhela S: Dolutegravir plus two different prodrugs of tenofovir to treat HIV. *N Engl J Med.* 2019;381(9):803–815. 10.1056/NEJMoa1902824 31339677

[28] Yah CS Tambo E Khayeka-Wandabwa C: Impact of telemonitoring approaches on integrated HIV and TB diagnosis and treatment interventions in sub-Saharan Africa: a scoping review. *Heal Promot Perspect.* 2017;7(2):60–65. 10.15171/hpp.2017.12 28326285 PMC5350551

[29] Knettel BA Mulawa MI Knippler ET: Women’s perspectives on ImpACT: a coping intervention to address sexual trauma and improve HIV care engagement in Cape Town, South Africa. *AIDS Care.* 2019;31(11):1389–1396. 10.1080/09540121.2019.1587368 30821168 PMC6717688

[30] Katz I Bogart L Courtney I: The treatment ambassador program: a pilot randomized controlled trial for people living with HIV in South Africa who delay or discontinue treatment. 13th International Conference on HIV Treatment and Prevention Adherence, Miami Beach, FL: June 8-10.2018 Reference Source

[31] Bengtson AM Chibwesha CJ Westreich D: A risk score to identify hiv-infected women most likely to become lost to follow-up in the postpartum period. *AIDS Care.* 2016;28(8):1035–1045. 10.1080/09540121.2016.1144869 26887526 PMC4974091

