# Supplementary material for: Models of service delivery for optimizing a patient’s first six months on antiretroviral therapy for HIV: an applied research agenda
Source: Gates Open Res. 2020 Jul 29;4:116. [Version 1] doi: 10.12688/gatesopenres.13159.1 (PMC7445417; doi:10.12688/gatesopenres.13159.1)
Supplement: Peer review history

# Peer review: Reviewer response for version 1

**DOI:** 10.21956/gatesopenres.14355.r29348

**Published:** 2020-08-28

**Authors:** Christopher J. Hoffmann

**Affiliations:** 1 Johns Hopkins University, Baltimore, MD, USA

Thank you for the opportunity to review the Open Letter “Models of service delivery for optimizing a patient’s first six months on antiretroviral therapy for HIV: an applied research agenda.” This commentary was written by leading researchers in the field following a Round Table that included prominent academicians and thinkers. The authors provided well written and well laid-out arguments for identifying and evaluating new approaches to improve care outcomes during the first 6 months after ART initiation. The justification for identifying effective approaches to improve early ART care are clear: loss from care is high during the first 6 months after ART initiation. Little work has specifically focused on adjusting the care model to overcome some of this loss from care. The authors make an effective argument that this period needs to be part of the care model conversation and included in focused research. Finally, the authors provide a list of research questions that, if answered, could help to inform new care models for this period of care. Overall this is a nice contribution to the discussion on improving early care outcomes.

 I have the following comments and suggestions:

1. Although the Round Table included leading researchers, mostly from the global north, the list of participants appeared to lack balance. The perspective of implementers on actual adaptation from some of the standard traditional practice may have added depth.
2. Some discussion and examples of adaptation from standard practice would be helpful for the reader to see what innovation is occurring. Innovation is suggested by the finding that 70% of differentiated care programs are for people who have completed at least 6 months of ART. This implies that 30% of differentiated care programs include components for people with <6m of ART. Describing some of these approaches would be interesting. Further adaptation has also occurred with COVID-19, with efforts to reduce clinic visits, even after ART initiation. Although these may not be part of an evaluation, some description of adaptation of early ART management to reduce clinic contact due to COVID-19 would be useful as well. This could also reinforce the need to understand what actually works best as change is occurring.  
3. The authors list internalized stigma (I assume HIV stigma and not shame from early loss from care) as an important reason for early loss from care. It would help to understand what new model of care could effectively address this issue. There are other reasons for early loss from care related to the cost (opportunity and financial) of repeat clinic visits, etc. that are easier to see how a change to care models could shift the balance from cost to value for a patient. It suggests adding a list of some of the reported causes of early loss from care which have been reported in multiple qualitative studies.
4. Figure 1 nicely illustrates the focus of this commentary and the gap in tested innovations in this space.
5. The priority research questions are all reasonable and presenting discrete questions is useful. However, I believe that as presented it isn’t clear that the there is a single (patient centered) goal to achieve at 6 months and subsequently. Ideally the metric for assessing these components fits into that goal and the other components of care for the first 6 months.  
6. It was interesting that psychosocial support was not clearly mentioned in the Table in light of internalized stigma being presented as the only example of reasons for early care disengagement. What, how, and to whom seems important in this regard (and has been the subject of study with peer supporters, digital support, and more traditional HIV support groups.

Does the article adequately reference differing views and opinions?

Partly

Are all factual statements correct, and are statements and arguments made adequately supported by citations?

Yes

Is the Open Letter written in accessible language?

Yes

Where applicable, are recommendations and next steps explained clearly for others to follow?

Yes

Is the rationale for the Open Letter provided in sufficient detail?

Yes

Reviewer Expertise:

HIV implementation science with a focus on optimizing care outcomes and survival for people with HIV in low and middle income countries.

I confirm that I have read this submission and believe that I have an appropriate level of expertise to confirm that it is of an acceptable scientific standard, however I have significant reservations, as outlined above.

# Peer review: Reviewer response for version 1

**DOI:** 10.21956/gatesopenres.14355.r29350

**Published:** 2020-08-25

**Authors:** Peter Bock

**Affiliations:** 1 Desmond Tutu Tuberculosis Centre, Department of Paediatrics and Child Health, Faculty of Medicine and Health Sciences, Stellenbosch University, Cape Town, South Africa

Many thanks for the opportunity to review this really important and well-written letter, some comments on the content below.

**Major comment:**

- Overall I think these research questions listed are very limited in scope and seem highly context specific - would there not be scope for a programmatic cluster trial that evaluated a package of care?

**Minor comments:**

- *As countries around the world strive to reach global targets for HIV, including starting and retaining 95% of those diagnosed with HIV on antiretroviral treatment (ART)* - Does this refer to UNAIDS targets – not sure if exactly correct? Please reference.
- E *ach of these three categories of patients is likely to have  distinct clinical and non-clinical needs during the first six  months. - *This is a good point but there is also an argument for problems with having too differentiated an approach at the busy interface between healthcare staff and clients. Also there is a risk of making these assumptions – albeit it is a good idea without unequivocal evidence so there is a balancing act that may be worth referring to? But I see you make these points well later on.
- I think the point you make about emphasising support for HIV positive patients with advanced disease is a good one and doesn’t require further evidence.

Does the article adequately reference differing views and opinions?

Partly

Are all factual statements correct, and are statements and arguments made adequately supported by citations?

Partly

Is the Open Letter written in accessible language?

Yes

Where applicable, are recommendations and next steps explained clearly for others to follow?

Yes

Is the rationale for the Open Letter provided in sufficient detail?

Yes

Reviewer Expertise:

HIV treatment and prevention

I confirm that I have read this submission and believe that I have an appropriate level of expertise to confirm that it is of an acceptable scientific standard, however I have significant reservations, as outlined above.

# Peer review: Reviewer response for version 1

**DOI:** 10.21956/gatesopenres.14355.r29239

**Published:** 2020-08-24

**Authors:** Richard Lessells

**Affiliations:** 1 KwaZulu-Natal Research Innovation & Sequencing Platform, College of Health Sciences, University of KwaZulu-Natal, Durban, South Africa

This open letter lays out the rationale for developing differentiated models of care for people starting antiretroviral therapy and in the first six months (not just people who are established on ART and stable), and includes a proposed research agenda to guide the development. This stems from a technical roundtable discussion held in early 2020 involving a number of clinicians, epidemiologists, implementers, health economists, advocates and funders involved in HIV treatment and care in sub-Saharan Africa.

 The authors make a strong argument why the development of innovations in this period are important, and it’s an argument that I certainly agree with. The priority research questions are sensible and pretty comprehensive. This is a good starting point to stimulate more ideas in this area and to help guide this research agenda.

 I have a few minor comments that could be considered by the authors:

1. Introduction, p3: On the fifth point that the COVID-19 epidemic has highlighted the potential risks of exposure to SARS-CoV-2 at healthcare facilities, I thought it might be worth pointing out here that of course we have also been aware for a long time of the risk of exposure to TB in healthcare facilities (for patients and healthcare workers) and have not done enough to mitigate this risk.
2. Table 1, Clinical requirements: I think an important point that is mentioned in the text but not really covered by any of the research questions is the integration between care delivery for HIV and other chronic diseases. Innovative models of HIV care in this time period will have limited impact for an individual if they still have to attend a clinic every month for their hypertension or diabetes medication. So understanding what people’s clinical needs are outside HIV care would be important. Was this discussed in the roundtable? Could there be a question framed around this issue?    
3. On a similar point around need for integrated care, for question 3 I think it might be worth highlighting the need to identify the main HIV-related and non-HIV-related reasons for healthcare visits.
4. Other considerations, p7: On the point about triaging patients to different levels of retention support at the time of ART initiation, the valid point is made that we have never identified consistent reliable predictors of poor retention. The statement is made that ‘Further research in this area may also be of value’. One could argue that if we haven’t found reliable predictors by now, we probably aren’t going to no matter how much research we do. So I think this statement could be strengthened by explaining why further research might be worthwhile – what would be different from all that has been done already?

I have one additional comment that does not relate directly to the manuscript but to the broader process. I note that, although all the authors and roundtable participants are all involved in HIV research in Africa, the majority (of authors and roundtable participants) are not from Africa but from North America and Europe. I hope that there is a commitment on the part of the authors, funders and other drivers of this research agenda for more active participation from African scientists, clinicians, implementers, and policy makers as this moves forward.

Does the article adequately reference differing views and opinions?

Yes

Are all factual statements correct, and are statements and arguments made adequately supported by citations?

Yes

Is the Open Letter written in accessible language?

Yes

Where applicable, are recommendations and next steps explained clearly for others to follow?

Yes

Is the rationale for the Open Letter provided in sufficient detail?

Yes

Reviewer Expertise:

HIV treatment and care, HIV epidemiology

I confirm that I have read this submission and believe that I have an appropriate level of expertise to confirm that it is of an acceptable scientific standard.

# Peer review: Reviewer response for version 1

**DOI:** 10.21956/gatesopenres.14355.r29354

**Published:** 2020-08-21

**Authors:** Tom Decroo

**Affiliations:** 1 Department of Clinical Sciences, Institute of Tropical Medicine, Antwerp, Belgium

With interest I read “Models of service delivery for optimizing a patient’s first six months on antiretroviral therapy for HIV: an applied research agenda”. The manuscript addresses an important topic and is very well written.

 Please find a first comment for a minor revision and a second comment for your consideration.

 “Because DSD models were originally conceived as a way to manage rapidly expanding populations of experienced patients who are clinically “stable” on ART”. The first documented DSD model was piloted to serve populations out of reach of the formal health system. Hence, DSD are not only about more efficient provision of care for stable patients, but also about differentiating care provision to meet with the reality of people’s daily life, also for those living in a rural community or those who experience stigma when visiting a health facility.

 I agree with the authors that the present facility-based model of care is not providing the best possible care for some patients, illustrated by the relatively high level of attrition during the first 6 months of treatment. Hence, this “gold standard” has its own limitations. Moreover, problems are underestimated as most data only report outcomes among those who sought health facility-based care. Those out of reach, or experiencing stigma, are not included in most study denominators. Given the limitations of the present gold standard there should be room for more drastic modifications than those proposed to test. While I agree that it is meaningful to compare the effect of each of the different components of the conventional facility-based care model with alternative approaches in community-based care, as shown in table 1, I also invite the authors to go one step further. Why not ask the opposite question? How can facility-based activities be adapted to support/complement community embedded and PLHIV network-driven comprehensive HIV care models?

 Please also consider adding questions related to self-efficacy and ownership, known drivers of sustained ART adherence. With comprehensive HIV care all activities (testing, ART initiation, early and long term ART) would be planned together with community members and provided within the community, while maintaining a strong link with the health facility. Instead of using criteria for referral from facility-based to DSD, patients with specific needs would be referred to a health facility, while continuing as a member of a social network. Of course, such an approach would not replace the facility-based approach, but complement it, for efficient management of stable patients, but also for those who experience barriers to facility-based care.

Does the article adequately reference differing views and opinions?

Yes

Are all factual statements correct, and are statements and arguments made adequately supported by citations?

Yes

Is the Open Letter written in accessible language?

Yes

Where applicable, are recommendations and next steps explained clearly for others to follow?

Yes

Is the rationale for the Open Letter provided in sufficient detail?

Yes

Reviewer Expertise:

community-based HIV care

I confirm that I have read this submission and believe that I have an appropriate level of expertise to confirm that it is of an acceptable scientific standard.
